# Morbidity associated with anterior iliac crest bone graft harvesting in children undergoing orthopaedic surgery: a prospective review

**DOI:** 10.1007/s11832-015-0698-0

**Published:** 2015-10-05

**Authors:** A. Clarke, M. J. Flowers, A. G. Davies, J. Fernandes, S. Jones

**Affiliations:** Department of Orthopaedics, Sheffield Children’s Hospital, Sheffield, UK; Department of Orthopaedics, Al Ahli Hospital, Doha, Qatar

**Keywords:** Anterior iliac crest bone graft, Morbidity, Children, Orthopaedic surgery

## Abstract

**Purpose:**

Autologous iliac crest bone grafting is an integral part of many orthopaedic surgical procedures. Several studies have documented morbidity and prolonged pain following iliac crest bone graft harvesting in adults; however, in children there is a paucity of information. The purpose of the present study was to quantify the degree of pain and morbidity associated with anterior iliac crest graft harvesting in children undergoing non-spinal orthopaedic surgery.

**Methods:**

Patients were prospectively enrolled prior to orthopaedic surgery. A patient self-reported visual analogue score was used to record pain at specified time points following surgery. In addition, the patients were reviewed at 2 and 6 weeks, 3 months and 1 year after surgery to record any complications.

**Results:**

Data was collected on 33 patients (34 graft sites). Only one patient (2.94 %) had a complication, namely an injury to the lateral femoral cutaneous nerve. This resolved 3 months after surgery. 89 % of patients had no pain at the iliac crest graft harvest site 3 months after surgery. The three patients who had pain at 3 months had visual analogue scores of 1.0, 1.1 and 1.3, respectively.

**Conclusion:**

This series reveals a very low complication rate and minimal iliac crest graft harvest site pain in children undergoing non-spinal orthopaedic surgery. In addition, the pain experienced is short-lived.

## Introduction

Bone grafts are used in orthopaedic surgical procedures to provide support, fill voids and promote healing. The iliac crest remains the preferred donor site when an autograft is used, as it provides good quantities of cortical and cancellous bone, is easy to access and possesses osteogenic, osteoconductive and osteoinductive properties [1, 2].

The morbidity associated with harvesting iliac crest bone grafts in adults is well documented [3, 4]. Potential complications include nerve damage, bleeding, infection, pelvic fractures, haematoma and sensory loss [5]. Several studies have also documented severe and prolonged pain at the iliac crest following surgery [2–4, 6]. In view of this, various alternatives to iliac crest autografts, including allografts, bone marrow aspirate, de-mineralised bone matrix and synthetic materials such as calcium hydroxyapatite, tri-calcium phosphate and bioactive glass have been used [7–10]. These do not match the effectiveness of the patient’s own bone and there are also cost implications to consider when using synthetic materials [11, 12].

In children there is a paucity of data. A few studies have reported the complications associated with harvesting iliac crest bone grafts for spinal procedures, although they used the posterior crest as a donor site, which has slightly different morbidities associated with it. In addition, it is more difficult to evaluate pain when the posterior iliac crest is used as the donor site in spinal surgery [4, 13–15].

Reports examining the anterior iliac crest have focused on facio-maxillary surgery [16, 17].

Furthermore, the majority of studies in both the adult and paediatric populations have been retrospective and do not provide a true representation of the complications and pain levels associated with harvesting anterior iliac crest bone grafting.

In view of this, we undertook a prospective evaluation of the morbidity associated with harvesting anterior iliac crest bone grafts in children undergoing non-spinal orthopaedic surgery.

## Materials and methods

After obtaining local ethics committee approval, we undertook a prospective review of all patients requiring anterior iliac crest bone grafting as part of their orthopaedic surgical procedure at the Sheffield Children’s Hospital between August 2012 and October 2013.

Patients who were due to have iliac crest bone grafting as part of their surgical treatment were identified from the surgical waiting list. The patients and their parents were then approached by one of us (AC) at the pre-operative assessment clinic and considered for entry into the study. Exclusion criteria included patients who were aged <7 years, had previous iliac crest graft harvesting, a history of congenital insensitivity to pain and learning difficulties.

Children aged <7 were excluded on the advice of the pain management team based on evidence that they would not be able to fill in the visual analogue scale (VAS) correctly. Out of 43 patients identified, 33 met the inclusion criteria and agreed to participate in the study. The patients consented after being provided with verbal and written information on the study. There were 11 females and 22 males with ages ranging from 7 to 16 years (mean 12 years).

A 10-cm VAS questionnaire was given to the patients on the morning of their surgery. They were expected to fill it in before surgery and then again at several specified time points afterwards, up to and including 3 months after surgery. The scale ranged from ‘0 = no pain at all’ to ‘10 = worst pain ever’. Two scales were provided for each time point: one for the hip and one for the recipient site (usually the foot). The anaesthetic administered and the medication for managing post-operative pain were similar for all patients.

The iliac crest bone graft was harvested utilising a skin incision along the ‘bikini line’ and a standard apophyseal splitting approach. The donor site was infiltrated with local anaesthetic and adrenalin prior to harvesting. The graft harvested depended on the indication. Wound closure was with deep dermal and sub-cuticular absorbable sutures. No drains were used.

Patient demographics including age, sex, body mass index (BMI), diagnosis, presence of co-morbidities, procedure requiring bone graft, type of graft harvested, length of iliac crest wound, intra-operative complications and length of hospital stay were recorded.

On discharge from hospital the patients were given a week’s supply of pain-relieving medication and were seen in the outpatient clinic at 2 and 6 weeks and 3 months after surgery. At these visits any complications and their progress were recorded. They were asked about the need for additional pain medication. Their questionnaires were collected for analysis after their 3-month review.

All 33 patients (34 iliac crest sites) recruited were available for review at the final follow-up, though only 27 questionnaires were fully completed, giving a response rate of 81.8 %. One patient had bilateral non-simultaneous surgery.

The results were analysed statistically using IBM SPSS Statistics for Windows, version 19.0. A Friedman analysis followed by post-hoc Wilcoxon signed-rank tests using Bonferroni-adjusted *p* values was used to compare the pain recorded over time to the baseline measurement.

The relationship between pain at the iliac crest and pain at the recipient site was also investigated using Pearson’s product moment correlation coefficient, following verification of assumptions.

## Results

The mean length of hospital stay in all patients was 1.5 days (range 1–5 days) and the mean length of the iliac crest wound was 4.88 cm (range 2.5–8.0 cm, SD = 1.38). All wounds had healed by the 2-week outpatient review.

Out of the 33 patients (34 iliac crest sites) included in the study, only one patient had a complication (2.94 %). This patient had numbness over the lateral aspect of the thigh, in keeping with lateral femoral cutaneous nerve (LFCN) injury. The numbness resolved 3 months after surgery.

In this patient, like all other patients, the graft was harvested using a standard apophyseal splitting approach through a ‘bikini-line’ incision over the iliac crest. The graft was required for a lateral column lengthening procedure to correct flat foot deformity.

**Fig. 1:**
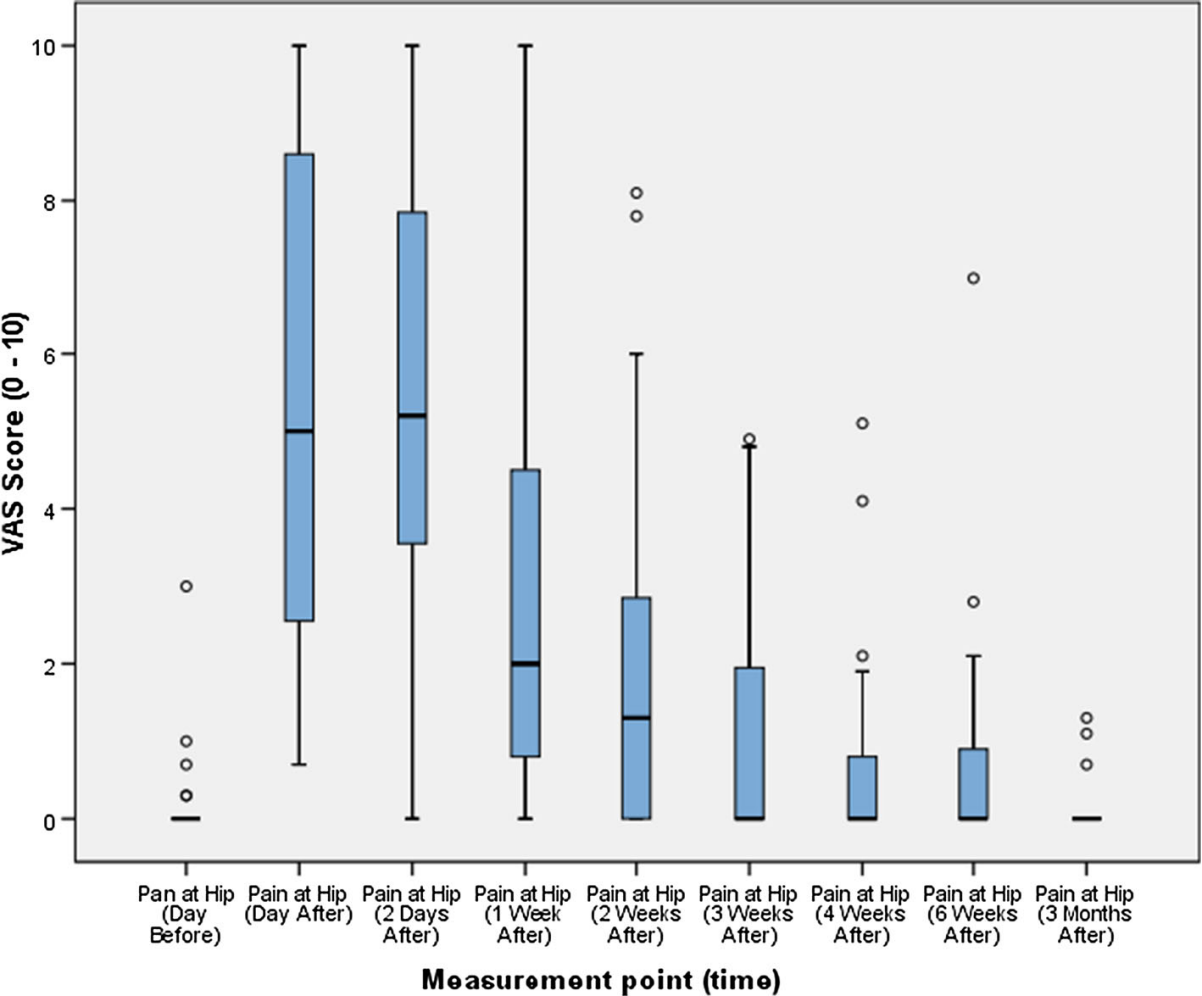
*Box* and *whisker* plot of VAS score for the iliac crest at respective time points

The VAS score at the iliac crest (donor site) increased from baseline before peaking 2 days after the operation and then decreasing. Figure 1 depicts the box and whisker plots for the VAS score at the iliac crest at respective time points. By 3 months post-operation, 24 patients (89 %) had recorded a VAS score of zero (‘no pain at all’). The remaining three patients recorded scores of 1.0, 1.1 and 1.3, respectively.

Further analysis was undertaken to provide a time to VAS = 0 or ‘no pain at all’. All patients who did not record a VAS score of 0 were censored at the 3-month point (11 % of patients). The results of the analysis showed a median time to VAS = 0 of 21 days (95 % confidence interval = 9.126–32.874).

Neither the results nor the residuals of the VAS scores at the various time points were normally distributed. A non-parametric Friedman test was therefore used to investigate the change in VAS score from the baseline to the different time points used in the study. The results of this analysis showed there was a statistically significant difference in VAS score for the iliac crest across the various measurement points: X^2^ (8, *n* = 27) = 161.907, *p* = 0.00.

Post-hoc tests were performed using Wilcoxon signed-rank tests between the baseline and all subsequent measurement points to examine where the differences lay. A Bonferroni adjusted *α* value was used (*p* = 0.00625) to control for Type 1 errors (Table 1).

**Table 1:** Friedman test for VAS score at the iliac crest using post-hoc Wilcoxon signed-rank tests with Bonferroni-adjusted *p* value

Wilcoxon signed-rank test comparison	*Z* value	*p* value (significance level = 0.00625)	Effect size (*r*)
Pain at hip (day after)–pain at hip (day before)	−4.54	0.000	0.62
Pain at hip (2 days after)–pain at hip (day before)	−4.46	0.000	0.61
Pain at hip (1 week after)–pain at hip (day before)	−4.11	0.000	0.56
Pain at hip (2 weeks after)–pain at hip (day before)	−3.74	0.000	0.51
Pain at hip (3 weeks after)–pain at hip (day before)	−2.90	0.004	0.39
Pain at hip (4 weeks after)–pain at hip (day before)	−2.06	0.039	0.28
Pain at hip (6 weeks after)–pain at hip (day before)	−1.69	0.091	0.23
Pain at hip (3 months after)–pain at hip (day before)	−0.42	0.671	0.06

The table shows there was no statistically significant difference between pain at the hip (iliac crest) at baseline and at 4 weeks after the operation (*Z* = −2.060, *p* = 0.039), 6 weeks after the operation (*Z* = −1.690, *p* = 0.091) and 3 months after the operation (*Z* = −0.424, *p* = 0.671).

A more detailed investigation of potential risk factors and their predisposition to iliac crest pain was undertaken. The factors investigated included gender, age, BMI, comorbidities, wound length, side of graft harvest, type of graft harvested, indication for bone graft and grade of surgeon. The only factor found to have an effect was the procedure (indication) for which the graft was harvested.

The procedures were grouped into three categories: flat foot procedure (*n* = 16), other corrective osteotomy and fixation (*n* = 4) and other lower limb procedures requiring bone grafting (*n* = 7). The mean VAS was significantly lower for patients undergoing corrective osteotomies and fixation compared to those undergoing a flat foot procedure or other lower limb procedure requiring a bone graft.

**Fig. 2:**
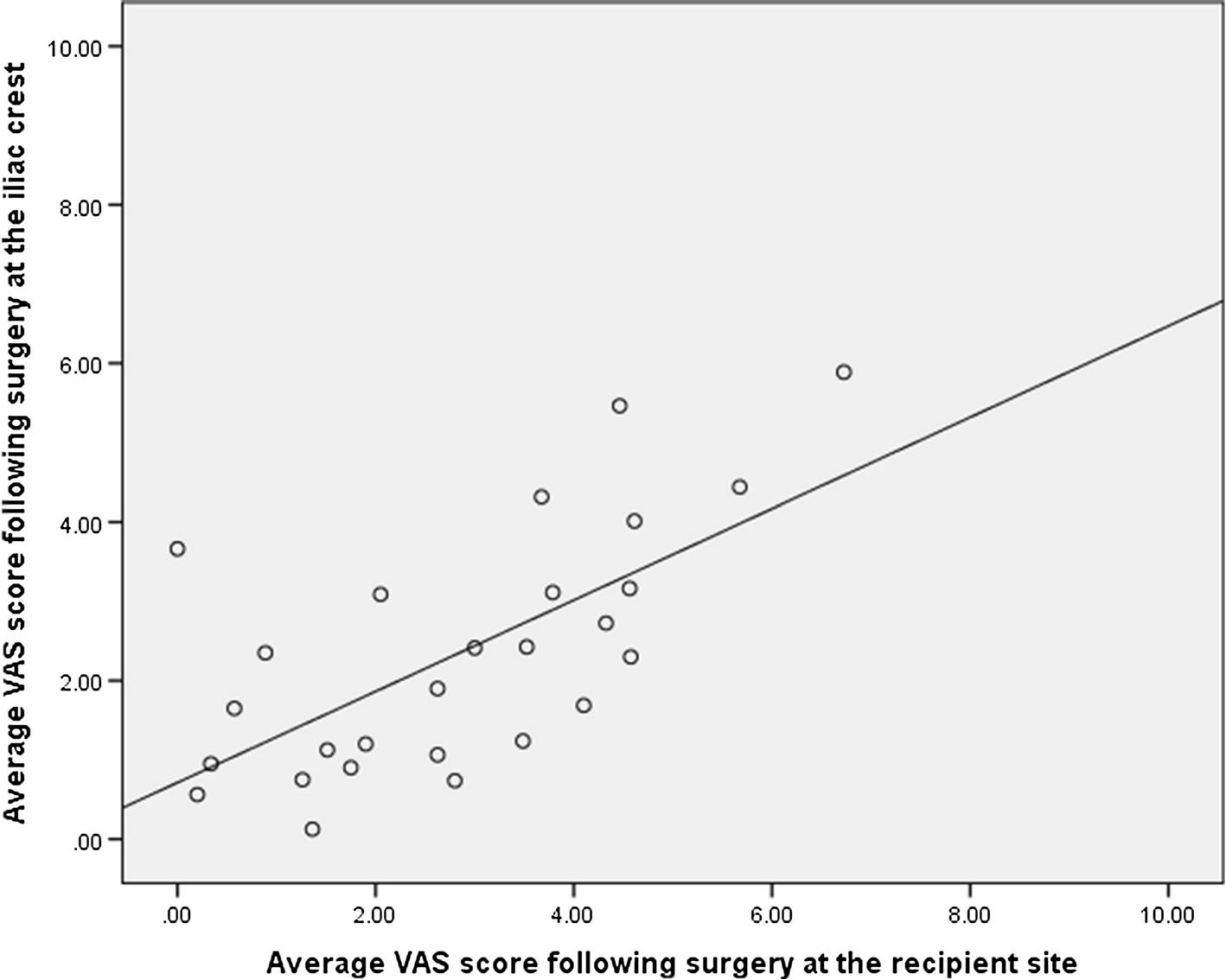
Scatter plot of mean VAS score at the iliac crest and mean VAS score at the recipient site following surgery

Finally, the relationship between the VAS score at the iliac crest and the recipient site was investigated using Pearson’s product moment correlation coefficient. A strong positive correlation was observed between the log of mean VAS score at the iliac crest and that at the recipient site (*r* = 0.662, *p* = 0.000) (Fig. 2).

## Discussion

The low complication rate of 2.94 % observed in this prospective review compares favorably to the rates of up to 24 % reported in the paediatric [13, 17] and 9.4−55 % in the adult population [2].

Skaggs et al. reported a complication rate of 24 % in a large retrospective review of 214 children undergoing spinal surgery using posterior rather than anterior iliac crest grafts [13]. The complications included arterial injury, infection, sacroiliac penetration and numbness. They also reported severe pain in 15 % of patients at the graft harvest site after a mean follow-up of 4 years. It is known that it can be difficult to differentiate donor from recipient site pain in patients undergoing spinal surgery who have grafts harvested from the posterior iliac crest [18] and this may account for the high pain incidence they reported. The advantage of our study is that all patients had a graft harvest site distinct from the recipient site, allowing a more accurate quantification of ‘true’ iliac crest/donor site pain.

Separate retrospective reports by Swan et al. and Burstein et al. on children undergoing alveolar cleft reconstruction using anterior iliac crest grafting observed complication rates of 6  and 9.5 %, respectively [17, 19]. The complications included superficial infection, haematoma, numbness and hypertrophic scars.

A recent systematic review reported 1249 (20 %) complications in 6449 adults requiring iliac crest bone grafts [2].

The rates of minor complications in the adult population are documented at between 6 and 39 %, while major complications are between 0.7 and 25 % [3–6]. No cases of infection, vascular injury, haematoma, extensive bruising, scar numbness, fracture or chronic pain were documented in the course of our study.

The incidence of lateral femoral cutaneous nerve (LFCN) injury has been reported to range between 1.7 and 31 % following iliac crest graft harvesting [6, 20–22]. Damage to this sensory branch, originating from the posterior roots of L2–3, can result in burning, tingling, numbness and/or pain over the anterolateral aspect of the thigh. A study of 205 cadaveric specimens found that 9.9 % of nerves had an aberrant course and were vulnerable to injury following anterior iliac crest graft harvesting [22].

Bierne et al. documented a 1.3 % incidence of LFCN injury in 137 patients (age 8–29 years). The low incidence led them to recommend the use of a ‘bikini-line’ incision (placing the skin incision 1 cm behind the anterior superior iliac spine) [23]. This measure, although sensible, is not infallible as the patient with this complication in our study had bone graft harvested using the ‘bikini-line’ incision. Other preventive measures include limiting the amount of soft tissue dissection and minimising stretching of the surrounding tissues.

The mean surgical scar length of 4.8 cm in our study is intermediate to that reported in other series using anterior iliac crest grafting. Cohen et al. reported a mean scar length of 4.0 cm, Swan et al. 6.0 cm and Laurie et al. 7.0 cm [17, 21, 24].

The visual analogue scale is a validated and commonly used pain scale, suitable for children aged 7 years and over [25–27], hence our choice of this scale.

The current study found that severe pain outside the immediate post-operative period was not common. Only three (11.1 %) patients had pain at 3 months and of these three none reported pain >1.3 on the VAS. No patient had pain 4 months after surgery. This is extremely favorable compared to other reports in the paediatric literature [13, 14, 17].

Retrospective reviews by Skaggs et al. noted that 24 % of patients reported pain up to 4 years after harvesting a posterior iliac crest graft for spinal surgery while Swan et al. observed that 7 % of their patients had pain that resolved 6 months after an anterior iliac crest graft was harvested [13, 17].

Though Kager et al. in a prospective review using posterior iliac crest graft for spinal surgery reported that none of the patients had a pain score of more than 3, they stated that 13 % had pain at 1 year, 6 % at 2 years and 12 % at 3 years [14].

Our results also suggest that paediatric patients are pain-free on average sooner than adult patients. Whilst none of the patients in our study had pain at 4 months, reports in the adult literature quote pain levels in 39 % of patients at 3 months, 38–42 % at 6 months and 19–21 % at 2 years after surgery [4, 18, 28].

The positive correlation between pain at the iliac crest and that at the recipient site could be due to the patient’s own perception of pain. It is known that during surgery pain signals generate a secondary inflammatory response, which contributes to post-operative pain. This so-called ‘spinal wind-up’ process can result in a state of post-operative hypersensitivity to pain, due to a combination of peripheral sensitisation due to surgical trauma and central sensitisation due to increased activity of spinal neurons. This could explain the findings of this study, as patients with an increased sensitivity to pain following surgery would be likely to report this at all sites [28, 29]. This reduction in pain threshold can be particularly resistant to analgesia, highlighting the need for pro-active pain management during iliac crest bone graft harvesting. This may also explain the higher levels of pain that have been documented in studies where the incision used to harvest the graft is close to the recipient site, such as in spinal surgery.

Inherent issues with the retrospective studies quoted mean that the results need to be interpreted with a degree of caution considering the potential impact of bias and other confounding factors. The prospective nature of the current study means that pain can be assessed more reliably without the recall bias that retrospective studies are prone to.

The limitations of this study are that the numbers are small and increasing the study size would increase confidence in its findings. Like all previous studies on this subject, the patient’s ability to accurately record pain is also a limitation, especially in younger patients, as pain is a subjective phenomenon that can only be characterised by the patient.

## Conclusion

The low post-operative pain level and complication rate of this study supports the continued use of anterior iliac crest bone grafts in children undergoing non-spinal orthopaedic surgery requiring bone grafting. In addition, the results of this study are a useful guide in the consenting process for bone graft surgery.
